# A Hypothesis: Hydrogen Sulfide Might Be Neuroprotective against Subarachnoid Hemorrhage Induced Brain Injury

**DOI:** 10.1155/2014/432318

**Published:** 2014-02-23

**Authors:** Yong-Peng Yu, Xiang-Lin Chi, Li-Jun Liu

**Affiliations:** ^1^Department of Neurology, Wendeng Center Hospital of Weihai, The Affiliated Hospital of Weifang Medical College, Weihai 264400, China; ^2^Department of Neurology, The Affiliated Hospital of the Medical College of Qingdao University, Shandong 266003, China

## Abstract

Gases such as nitric oxide (NO) and carbon monoxide (CO) play important roles both in normal physiology and in disease. Recent studies have shown that hydrogen sulfide (H_2_S) protects neurons against oxidative stress and ischemia-reperfusion injury and attenuates lipopolysaccharides (LPS) induced neuroinflammation in microglia, exhibiting anti-inflammatory and antiapoptotic activities. The gas H_2_S is emerging as a novel regulator of important physiologic functions such as arterial diameter, blood flow, and leukocyte adhesion. It has been known that multiple factors, including oxidative stress, free radicals, and neuronal nitric oxide synthesis as well as abnormal inflammatory responses, are involved in the mechanism underlying the brain injury after subarachnoid hemorrhage (SAH). Based on the multiple physiologic functions of H_2_S, we speculate that it might be a promising, effective, and specific therapy for brain injury after SAH.

## 1. Introduction

Nitric oxide (NO) and carbon monoxide (CO) are established physiologic messenger molecules, and the former serving as an endothelial cell-derived relaxing factor (EDRF) has an important role in the regulation of blood pressure [1]. Even though hydrogen sulfide (H_2_S) has long been known as a noxious and toxic gas, recent accumulated evidence suggests that H_2_S, as an important endogenous vasodilator and neuromodulator [2, 3], has been implicated in similar functions [4]. A physiologic role for H_2_S in regulating blood pressure, its potent neuroprotective [5], and anti-inflammatory effects support a hypothesis that H_2_S might act as an effective agent that may have therapeutic potential against brain damage induced by oxidative stress, inflammation, hypoxic vasoconstriction, and other factors. Brain damage mainly induced by cerebral vasospasm is a potentially incapacitating or lethal complication in patients with aneurysmal SAH. Thus, the development of effective preventative and therapeutic interventions is an urgent and significant need. The objective of this paper is to present an overview of the pathogenesis of brain injury after SAH and the multiple physiologic functions of H_2_S in the vascular system, based on which a hypothesis is providing that H_2_S might be an effective therapy agent for brain injury after SAH.

## 2. Distribution and Level of H_**2**_S in Vascular Tissues

There are three known enzymes that produce H_2_S endogenously in mammalian tissue: cystathionine-synthase (CBS), gamma lyase (CGL or cystathionine gamma-lyase, CSE), and 3-mercaptopyruvate sulfur transferase (3MST). There are three major fates of H_2_S in the body. First, most of the H_2_S produced in the body is oxidized in the mitochondria to an end product of sulfate. The remaining H_2_S either is methylated by thiol S-methyltransferase (TSMT) to methanethiol and dimethyl sulfide or binds to methemoglobin to form sulfhemoglobin. In most tissues, CBS and CSE, which are responsible for catalyzing the production of H_2_S, are both pyridoxal-5-phosphate-dependent enzymes that utilize cysteine and homocysteine as substrates to liberate ammonium, pyruvate, and H_2_S [6]. It was originally believed that CBS was responsible for H_2_S production in the brain through the activation of the Ca^2+^/calmodulin pathway. H_2_S is produced by 3MST from l-cysteine and alpha-ketoglutarate through the metabolism with cysteine aminotransferase (CAT) [7].

H_2_S production was observed in ileum, portal vein, and thoracic aorta homogenates when L-Cys and PLP were administered. Moreover, the application of aminooxyacetate (CBS inhibitor) inhibited H_2_S production in ileum but failed to affect generation of H_2_S in portal vein and thoracic aorta, suggesting the lack of CBS in vascular tissues [8, 9]. Many studies have been focused on this issue [3, 10–13]. Endogenous H_2_S has been paid more and more attention to since its physiological discovery. It is expected that physiological concentration of H_2_S may vary extensively from synthesizing enzymes in different tissues. It was reported that endogenous concentration of H_2_S in rat, human, and bovine brains ranged within 50–160 *μ*M [14–16], whereas its serum concentration was about to be 50 *μ*M. Previous study showed that H_2_S does not circulate in the plasma at high enough concentration to be detectable in blood and plasma from a variety of animals, including trout, mouse, Wistar rat, Dawley rat, pig, and cow [11]. H_2_S concentration in brain and liver homogenates was measured 14 ± 3 nM and 17 ± 3 nM, respectively [12].

## 3. Multiple Physiologic Functions and Beneficial Effects of H_**2**_S in Biological Systems

The biological effects of H_2_S on vascular system have been studied for more than a decade. It was first reported that H_2_S concentration dependently relaxed norepinephrine precontracted portal vein and thoracic aorta, and this relaxation effect was reversible upon removal of chemicals [8]. This vasodilatory effect was later found to be present not only in thoracic aorta, but also in other types of vascular tissues including mesenteric arteries, pulmonary artery, and tail artery. H_2_S induced vasorelaxation is mainly brought about by the opening of K_ATP_ channels [17, 18]. Other signaling mechanisms for the vasorelaxant effect of H_2_S may involve depletion of intracellular ATP levels in aortic rings [19] and intracellular acidosis [20]. K_ATP_ channels are likely not to be involved in H_2_S induced vasoconstriction [21]. However, many studies were performed to attempt to unveil the underlying mechanism of H_2_S induced constrictive effects. Firstly, H_2_S may react with NO to form a compound, which by itself has no effect on vascular contractility. NO, as a potent physiological vasodilator, quenched by H_2_S, which might underlie H_2_S  induced vascular constriction response [19, 22]. Moreover, H_2_S exerted inhibitory effects on endothelial nitric oxide synthase (eNOS) activity [18]. H_2_S was found to downregulate cAMP levels in vascular smooth muscle cells (SMC) [21]. H_2_S exerts antihypertensive effects to different extents in different hypertensive models. The antihypertensive role of H_2_S is confirmed. Slow H_2_S-releasing compounds and therefore might be as potential therapeutics for hypertension treatment in the future. The data mentioned above suggests that the evidence for H_2_S's effects on vascular smooth muscle tone is contradictory.

The mechanisms by which H_2_S affects injured cells are complicated. Interestingly, in some studies, H_2_S induced proinflammatory effects which indicates that the background of these immunomodulatory influences still remains elusive [19]. H_2_S has several effects on mitochondria of cardiac cells such as the reversible inhibition of cytochrome C oxidase, which leads to preservation of mitochondrial structure and function after ischemia/reperfusion. Inhibition of mitochondrial respiration in the injured myocytes results in attenuated generation of reactive oxygen species (ROS) and may alter the function of the affected cell [22]. H_2_S decreases lipid peroxidation by scavenging hydrogen peroxide and superoxide in a model of isoproterenol induced myocardial injury. Besides these mechanisms, H_2_S also acts as a direct scavenger neutralizing cytotoxic reactive species like peroxynitrite.

The physiological actions of H_2_S make this gas ideally suited to protect the heart, brain, liver, kidney, and lungs against injury during ischemia/reperfusion (I/R). In recent years, the cytoprotective effects of endogenous and exogenous H_2_S have been investigated in models of in vitro and in vivo ischemic injury [6]. Previous study showed that either endogenous or exogenous increases in H_2_S at the time of reperfusion limit the extent of myocardial infarction, which was accompanied by a decrease in myocardial inflammation and mitochondrial function preservation [23]. Regulation and cytoprotective, physiological, and chemical roles of H_2_S in biological systems including ischemic injury of cardiac-cerebral vascular disease have been investigated in many studies [23–30].

The vasodilatory effect of H_2_S, which might be a potential candidate to be considered as EDRF and was later found to be present in widespread arteries including thoracic aorta, mesenteric arteries, pulmonary, and other types of vascular tissues [30], was particularly involved at the microcirculation level [31, 32]. Based on the accumulated data, several signaling pathways including cyclic adenosine monophosphate (cAMP)/protein kinase A (PKA), NO/cyclic guanosine monophosphate (cGMP)/protein kinase G (PKG) [33, 34], and calcium/calmodulin (CaM) [35] were involved in the vasoregulatory effect of H_2_S. Previous study also suggested that hypoxia and H_2_S shared a common and unique pathway in the excitation-contraction process. The inhibition of H_2_S synthesis can inhibit both hypoxic vasoconstriction and hypoxic vasodilation [36].

This role of H_2_S on smooth muscle is likely to be a general effect. Besides the recently established vasoregulatory role of H_2_S, its anti-inflammatory effect in different systems has been investigated. Recent studies have demonstrated that H_2_S may in fact limit inflammation and free radical damage [37], while another study indicated that H_2_S might exert an important proinflammatory role in regulating the severity of pancreatitis and associated lung injury [36]. Previous data suggested that ROS were implicated not only in the control of vascular tone in blood, but also in the H_2_S-induced regulatory function of vascular tissues [33, 38]. These findings raise the possibility that pharmacologic enhancement of H_2_S formation could be an alternative approach for repairing neuron damage induced by vasospasm, ischemia, oxidative stress, inflammation, and other factors, while previous study has demonstrated that H_2_S treatment improved endothelium-dependent coronary microvascular relaxation, providing biochemical myocardial protection via attenuation of caspase-independent apoptosis and autophagy in the experimental model [39]. It suggested that the evidence for H_2_S's effects on autophagy might be contradictory.

## 4. Causes and Mechanisms of Brain Injury after Subarachnoid Hemorrhage

Accumulated data indicate that not only delayed ischemic injury [40], which had been considered the most important cause of poor outcome after SAH, but also early brain injury has become the vital determinant of the intensity of later developing neurological complications [41]. Delayed cerebral vasospasm that develops 3–7 days after SAH had traditionally been considered the most important determinant of delayed ischemic injury. In recent years, increasing evidence suggests that several mechanisms, including ionic and physiological, biochemical, molecular, persistent vascular changes, cell death, oxidative stress, and inflammatory cascade activation, were involved in the pathogenesis of early brain injury after SAH [42–44]. Previous papers show that many experimental studies as well as autopsies performed on the brains of patients after SAH demonstrated extensive ischemic damage. Many factors including elevation of intracranial pressure (ICP), release of vasoactive substances during erythrocyte lysis, platelet aggregation, lipid peroxidation, unopposed sympathetic activity, and alterations in the nitric oxide/nitric oxide synthase (NO/NOS) pathways may contribute to this brain injury [45].

Though cerebral vasospasm probably plays some part in brain injury after SAH, neurologic injury may not be entirely explained by ischemia. The relationship between cerebral vasospasm and neurologic outcome might be associated with other coexisting causative factors such as microvascular dysfunction and complex neuronal-glial interactions. Previous study suggested that cerebral infarction contributed to poor outcome by vasospasm-independent effects after SAH [44, 46]. Cortical spreading depression (CSD) as one of the interesting mechanisms in brain damage after SAH has gained increasing attention [47, 48]. It has been previously reported that leukocyte-endothelial cell interactions, which played a significant role in the pathophysiology of cerebral vasospasm, could explain the clinical variability and time course of this disease [49]. Therefore, timely therapeutic targeting of the inflammatory response may prevent vasospasm-related brain damage and improve outcomes in patients with SAH.

Autophagy, being a self-degradative process, which plays a housekeeping role in removing misfolded or aggregated proteins, clearing damaged organelles, and eliminating intracellular pathogens [50, 51], is important for balancing sources of energy at critical times in development and in response to nutrient stress. The autophagy pathway has been reported to be involved in several central nervous system diseases such as cerebral ischemia [52], hypoxia-ischemia induced brain injury, and traumatic brain injury [53]. In the experimental SAH model, it is suggested that autophagy activation could participate in the pathogenesis of early brain injury induced by SAH. That is to say, activation of the autophagy pathway may play a potential role to attenuate the development of brain damage in SAH [51].

## 5. Involvement of H_**2**_S in Vascular Relaxation

H_2_S, known as a poisonous and toxic gas of the rotten egg, is endogenously produced from the metabolism of l-cysteine by constitutively expressed enzymes, including CBS and CSE [54, 55]. H_2_S produced by CSE can enhance outward flux of K^+^ via opening K_ATP_ channels, resulting in hyperpolarization of membrane potential and vascular smooth muscle relaxation [17, 56]. The vascular effects of H_2_S are suggested to be partially mediated by a functional endothelium [57]. It is also suggested to regulate smooth muscle tone in coordination with NO [8]. Previous study reported that CSE gene knockout mice displayed significant hypertension and decreased endothelium-dependent vasorelaxation, being accompanied by reduction of H_2_S levels in many tissues, including serum, heart, and aorta [58]. Intravenous delivery of NaHS, an H_2_S donor, can transiently decrease systolic blood pressure of mice, suggesting that H_2_S plays a vital role in regulating physiologic vasodilation and blood pressure.

The effect of H_2_S on regulating vascular smooth muscle tone and blood pressure implies that it might involve in regulating cerebral blood flow cerebral vasospasm after SAH and there is a possibility of its potential therapeutic effect on prevention and reversal of cerebral vasospasm. While the evidence on H_2_S associated with cerebral vasospasm after SAH is limited, more studies should be performed from the basic research in the future.

## 6. The Effect of NO in Pathophysiology, Reversal of Delayed Cerebral Vasospasm

NO, as the most well-known EDRF, is produced by eNOS in the intima and by neuronal nitric oxide synthase (nNOS) and inducible nitric oxide synthase (iNOS) in the adventitia of cerebral vessels and smooth muscle cells [59, 60]. Other substances which display properties in common with the characteristics of EDRF include CO [61] and H_2_S [62]. H_2_S, NO, and nitrite are formed in vivo and are of vital importance in the tissue response to hypoxia. These signaling molecules are involved in a multitude of processes including the regulation of vascular tone, cellular metabolic function, and cytoprotection [63].

NO can directly activate soluble guanylcyclase (sGC) to catalyze the conversion of GTP to cyclic GMP in smooth muscle cells. Increased levels of cyclic GMP lead to prevention of Ca^2+^-dependent activation of myosin light-chain kinase [64]. Activation of Ca^2+^-dependent K_ATP_ channels [65, 66] has been suggested to be involved in NO-induced vascular smooth muscle relaxation. In addition to causing vasodilation, decreasing vascular resistance, and lowering blood pressure, NO derived from endothelial cells can also inhibit platelet aggregation and adhesion and reduce smooth muscle proliferation [67].

In animals subjected to SAH, oxyhemoglobin (oxyHb) gradually released from blood clots in the subarachnoid space via erythrocyte lysis is a powerful scavenger of NO [68]. Together with vasoconstricting factors such as endothelin-1 (ET-1), cyclooxygenase (COX) products, and ROS [69], the decreased bioavailability of NO derived from endothelium, neurons, and nitrergic nerve would lead to cerebral vasospasm after SAH. Excessive production of NO plays a role under pathological conditions such as inflammation and cerebral ischemia, leading to generation of peroxynitrite and other highly toxic compounds. This may account partly for the delayed ischemic neurological deficit after subarachnoid hemorrhage. Also, previous studies have demonstrated that iNOS inhibition might yield therapeutic methods to alleviate ischemic brain injury [70]. On the other hand, preconditioning mediated by eNOS might have beneficial effects on reducing vasospasm and cerebral ischemia after SAH [71]. Thus, based on these data, the key therapeutic target in cerebral vasospasm after SAH would be exogenous administration of NO donors, inhibition of PDE and BOXes, and prevention of oxyHb neurotoxicity [72]. In animal models, intravenous administration of nitroglycerin (NTG) or sodium nitroprusside (SNAP) with NO in the form of nitrates could prevent cerebral vasospasm effectively [73, 74]. Nitrite, which is reported as an endogenous NO donor in blood [75], may provide a way to overcome reduced NO production in the arterial wall after SAH [72]. In a primate SAH model, intravenous continuous delivery of sodium nitrite for two weeks prevents development of vasospasm without changes in blood pressure, which demonstrates that nitrite could release NO locally in the subarachnoid space [76]. However, further study on nitrite needs to be done to elucidate pharmacokinetics of sodium nitrite in humans and establish proper dosage and safety profile. Regional (intracarotid/intracerebral arterial) or local (intraventricular or intrathecal) delivery of NO donors is not clinically attractive because of increased risk of severe complications or surgical access [72].

## 7. The Parallels and Contrastive Effects between NO and H_**2**_S: Looking for Crosstalk

NO and H_2_S are ancient, prebiotic, and chemically reactive molecules, which living organisms have evolved to cope with and ultimately make use of for signaling purposes. The reactivity of NO and H_2_S is controlled partly by keeping their steady-state levels in vivo rather low (in the nanomolar to low micromolar range) by balancing consumption with production, by the NOS enzymes in the case of NO and by the enzymes CSE, CBS, or the tandem enzymes cysteine aminotransferase (CAT) and 3-mercaptopyruvate sulfur transferase (3-MST) in the case of H_2_S. Another common feature of these enzymatic pathways is that both have an amino acid as substrate: L-Arg is the substrate for NOS and L-Cys is a substrate for CSE and CBS [63]. In the vertebrate circulation, NO and H_2_S diffuse away from their site of production and react specifically with their biological targets. NO may activate soluble guanylate cyclase present in adjacent smooth muscle cells and trigger vasorelaxation or react with ROS to form secondary reactive nitrogen oxide species (e.g., peroxynitrite). Conversely, H_2_S may activate potassium channels and, depending on whether the vessel is systemic or pulmonary/gill, may then trigger vasorelaxation or vasoconstriction, respectively, or even both at different time scales [77]. Thus, whereas NO is strictly a vasodilator, H_2_S may mediate dilation and/or constriction. The prevailing view is that both NO and H_2_S have a limited radius of effects and have mainly paracrine/autocrine actions. NO, however, can be transported in the blood as plasma and erythrocyte nitrite and S-nitrosothiols, which are relatively stable oxidation products of NO metabolism. Such products may then be recycled back to NO at distant locations and therefore NO can be also regarded as an endocrine signaling molecule. Such endocrine function has not been demonstrated for H_2_S up to now [63]. Furthermore, it is not yet clear to which extent H_2_S is capable of generating S-nitrosothiols or other related products. It appears conceivable that a possible crosstalk between the two pathways might involve some thiol-based biochemical compounds common to either pathway [78]. On the other hand, H_2_S relaxations are reported to be independent of NO synthesis or cGMP [79], which suggests other downstream targets specific for H_2_S. The antithrombotic effect of hydrogen sulfide is partly mediated by an upregulation of nitric oxide synthases [80]. Previous study reported that H_2_S affects [Ca^2+^]_*i*_ homeostasis which is mediated by H_2_S-evoked NO production [81]. These hypotheses, while pointing to a possible delicate balance between these two pathways, need to be confirmed experimentally.

Clearly, understanding the crosstalk between these two pathways is a major challenge for future investigations. Whereas highly sensitive and specific reductive chemiluminescence has been essential to determine type, concentrations, and fate of NO metabolites, concerning H_2_S detection in vivo, intracellular H_2_S concentrations are not known due to the lack of a suitable, sensitive, and specific method to analyze its various forms currently. As it is the case for NO that can be regenerated from nitrite, there is no evidence that H_2_S metabolites can be transported in the blood, stored in tissues, and reactivated to generate H_2_S at present. It is possible therefore that our understanding on this aspect may change with the development of alternative methodologies in the future. There is a need for future research in this field to better understand the complexity of biological interaction between the H_2_S and NO/nitrite signaling pathways.

## 8. H_**2**_S: A Novel Neuroprotectant for Central Nervous System Diseases Based on Its Role in Physiology

Since 1996, when H_2_S was firstly reported, the role of H_2_S has been gradually revealed by various contributions worldwide [82]. H_2_S has been generally recognized as an important signaling molecule in cardiovascular and nervous systems. The physiological effects of H_2_S has been confirmed by understanding the therapeutic implications of H_2_S in central nervous system (CNS) related diseases including neurodegenerative diseases, diabetes, and cancer, among others. In the CNS, H_2_S ameliorates ischemic injuries but leads to the aggravation of stroke. H_2_S concentration enhancement could induce cerebral infarct, while CBS or CSE inhibitors can reverse this effect on the brain. The role of H_2_S in neuron protection has been shown in glutamate-induced death with enhancement of cysteine and *γ*-glutamylcysteine concentrations, which leads to increasing concentrations of GSH [83–86].

Parkinson's disease (PD) is a common neurodegenerative disease with various manifestations, among which is cognitive deficiency, namely, dementia, which is characterized by progressive loss of dopaminergic neurons in the substantia nigra (SN). In a PD rat model induced by 6-hydroxydopamine (6-OHDA), the endogenous H_2_S level significantly decreased in the SN. However, H_2_S treatment can specifically inhibit accumulation of proinflammatory factors in the striatum and 6-OHDA-evoked NADPH oxidase activation, oxygen consumption, and microglial activation in the SN [87]. Dementia is usually ascribed to changes in the nucleus basalis of Meynert and the cerebral cortex [88]. H_2_S has also been suggested to attenuate vascular dementia injury via inhibition of apoptosis by regulating Bcl-2 and Bax expressions [89].

Alzheimer's disease (AD) is the most common form of dementia, which is pathologically characterized by the accumulation of senile plaques containing activated microglia and amyloid beta peptides (A-beta) [90]. As mentioned above, endogenous H_2_S is predominantly produced in the brain from cysteine by CBS. In the brains of AD patients, lower levels of H_2_S are a strong risk factor for the development of AD [91, 92]. Localized increases in H_2_S could delay aggravation and exacerbation of symptoms in patients with AD [85, 93, 94]. In addition, patients with Down syndrome overproduce H_2_S due to high level of the urinary excretion of thiosulfate, suggesting a positive relationship between H_2_S concentration and the aggravation of this disease [94–96]. Accordingly, it has been demonstrated that H_2_S has protective effects against A-beta-induced cell injury by inhibiting inflammation, promoting cell growth, and preserving mitochondrial function [97]. Moreover, H_2_S can protect neurons from oxidative stress, which is responsible for neuronal damage and degeneration in AD. H_2_S protects neurons against glutamate-mediated oxidative stress by enhancing the activities of *γ*-GCS and cystine transport, which results in increasing glutathione levels [98]. Neurotoxicity of elevated Hcy is associated with inhibition of endogenous H_2_S generation. It has been suggested that H_2_S could reduce neurotoxicity induced by Hcy and that enhancement of H_2_S synthesis may be a useful therapeutic strategy against Hcy-induced AD [99]. These lines of evidences suggest that H_2_S is a promising therapeutic target for treating neurodegenerative diseases.

## 9. Hypothesis and Theoretical Aspects Fundamental to This Hypothesis

At present, the mainstay treatment of brain injury after SAH is neurocritical care management aimed at reducing secondary brain injury, oral nimodipine, hemodynamic therapy, statin/magnesium/nicardipine therapy, cerebrospinal fluid (CSF) drainage, and endovascular techniques to improve cerebral vasospasm. Most of these treatments are aimed at one or two physiological and pathological mechanisms of brain injury after SAH. Then a therapy or therapies focused on multiple mechanisms may prevent the brain injury and improve the long-term outcome of SAH better. In fact, the possible roles and target of endogenous H_2_S in pathophysiological regulation of SAH have not been investigated. In light of the multiple physiologic roles and beneficial effects of H_2_S in mammalian tissue mentioned above, our hypothesis is that H_2_S might act as an effective agent which might provide a novel approach to the treatment of brain injury after SAH. This hypothesis is based on the following facts: it is suggested that vascular contractility is regulated by endogenous and exogenous H_2_S at physiologically relevant concentrations [17]. Furthermore, some studies reported that vasorelaxation response elicited by H_2_S was greater in small mesenteric arteries as compared to that in larger vascular tissues such as aorta [100]. H_2_S is an endogenous substance that is also produced, reaching an endogenous level of 50–160 *μ*M. Cerebral vascular smooth muscle cells would be exposed to significant amounts of H_2_S [58, 101]. Previous study indicated that exogenous H_2_S, generated as sodium sulfide, could limit the inflammatory response to acute myocardial I/R injury in an animal model [102].

It has been known that the mechanism underlying the brain injury after SAH is interlaced with multiple causative and/or pathogenic factors, including free radicals reactions, inflammatory processes, apoptosis, an imbalance between vasoconstrictor and vasodilator substances (endothelium derived substances, NO, endothelin, arachidonic acid metabolites, etc.), an upheaval of factors which regulate vascular tone, and endothelial proliferation [103]. As mentioned above, autophagy could participate in the pathogenesis of brain injury induced by SAH, which will provide novel ideas for pursuing therapeutic agents for SAH-induced brain injury. The success of these current therapies in reducing incidence of cerebral vasospasm without reduction in brain injury and improved quality of life indicates that treating vasospasm alone may not achieve favorable result. Therefore, therapies against SAH designed to direct towards inhibiting brain injury may prove more beneficial in preventing neurological deterioration.

## 10. Conclusion

In light of a wide range of physiological roles of H_2_S as mentioned above, it produces physiological and pathological functions in many organs and systems. Previous papers have reported that H_2_S could protect against reperfusion injury, lethal hypoxia, and exerted anti-inflammatory and antiapoptotic activities and oxidative stress effects [104] as well as effect of autophagy activation. Furthermore, as an endogenous gasotransmitter and a potential treatment, H_2_S has distinct advantages over pharmaceutical drugs: its tissue compatibility and high blood brain barrier permeability are stronger than many other antioxidants and it is an endogenous substance. We speculate that H_2_S could be a potentially effective approach to the treatment of brain injury including vasospasm after SAH. With our existing knowledge about the beneficial effects of H_2_S, the future of H_2_S as a potential therapy against brain injury after SAH is deemed to be promising and exciting.

## 11. Looking Forward: Challenges for Translation of the Toxic Molecule H_**2**_S and Its Therapeutic Application

Investigation of the role of H_2_S is still in its infancy. H_2_S has both scientific and technological values. The latter tends to dominate because of its financial value and direct applicability. The scientific investigation of H_2_S is also important for the elucidation of its base fundamental roles. Certainly, problems and challenges will arise and the limitations of experimental materials will constrain further research in the field of H_2_S. A sustained and controlled H_2_S-releasing donor that functions both in vitro and in vivo has not been found yet. With an increased understanding of the various H_2_S mechanisms in the body, further study of H_2_S becomes more difficult and complicated. H_2_S is known as a third gaseous signaling molecule, which means that it plays the role of a messenger. The concentration of H_2_S has been proven to have relevance in particular diseases; for instance, it is overproduced in sepsis and found at inadequate levels in AD [105]. Therefore, the mechanism controlling the actual concentration of H_2_S in certain tissues may become the ultimate problem for H_2_S related research. It should be emphasized that a relevant relationship does not mean a relationship of causation. By regulating the H_2_S concentration in particular tissues, symptoms of a specific disease can be controlled, which implies that the origin of the disease has not been addressed. That is to say, the regulation of H_2_S can only provide transient protection from certain diseases, such as hypertension. The challenges of the sustained and controlled release of H_2_S-releasing drugs were mentioned above. Another difficulty in H_2_S related research comes from the multiple functions of H_2_S, which cause a shortage of specific effects which are dose-, time-, and tissue-dependent.

The pathophysiology of cerebral vasospasm after SAH is complicated and needs to be further clarified. The role of NO in the regulation of cerebral blood flow, pathogenesis, and treatment of cerebral vasospasm and delayed ischemic neurological deficit after SAH is mostly investigated. Studies on other EDRFs such as H_2_S associated with cerebral vasospasm are limited and may provide potential targets for possible development of preventive and therapeutic measures on regulation of cerebral blood flow and brain injury after SAH including cerebral vasospasm. Interaction among these factors also needs to be investigated. The inflammatory response accompanying SAH may represent a crucial pathway in the pathogenesis of cerebral vasospasm and delayed ischemic neurological deficit [106, 107]. Experimental and clinical research is required to elucidate the role of EDRFs such as H_2_S in regulation of cerebral blood flow and cerebral vasospasm, the interventional methods for prevention and reversal of vasospasm, and protection from neurological ischemic deficits. For patients with different diseases, H_2_S may need to be administered as different drugs. Therefore, a focus on the general effects of H_2_S, such as on brain injury after SAH, is rational.
