# Corrigendum to “A Hypothesis: Hydrogen Sulfide Might Be Neuroprotective against Subarachnoid Hemorrhage Induced Brain Injury”

**DOI:** 10.1155/2020/8364250

**Published:** 2020-09-27

**Authors:** Yong-Peng Yu, Xiang-Lin Chi, Li-Jun Liu

**Affiliations:** ^1^Department of Neurology, Wendeng Center Hospital of Weihai, The Affiliated Hospital of Weifang Medical College, Weihai 264400, China; ^2^Department of Neurology, The Affiliated Hospital of the Medical College of Qingdao University, Qingdao, Shandong Province 266003, China

## Abstract

Gases such as nitric oxide (NO) and carbon monoxide (CO) play important roles both in normal physiology and in disease. Recent studies have shown that hydrogen sulfide (H_2_S) protects neurons against oxidative stress and ischemia-reperfusion injury and attenuates lipopolysaccharides (LPS) induced neuroinflammation in microglia, exhibiting anti-inflammatory and antiapoptotic activities. The gas H_2_S is emerging as a novel regulator of important physiologic functions such as arterial diameter, blood flow, and leukocyte adhesion. It has been known that multiple factors, including oxidative stress, free radicals, and neuronal nitric oxide synthesis as well as abnormal inflammatory responses, are involved in the mechanism underlying the brain injury after subarachnoid hemorrhage (SAH). Based on the multiple physiologic functions of H_2_S, we speculate that it might be a promising, effective, and specific therapy for brain injury after SAH.

The article titled “A Hypothesis: Hydrogen Sulfide Might Be Neuroprotective against Subarachnoid Hemorrhage Induced Brain Injury” [1] was found to contain material from other published articles, including the following sources. Therefore, the article has been revised as follows:

[2] Meng Qi. “Involvement of endothelial-derived relaxing factors in the regulation of cerebral blood flow,” Neurological Sciences, 05/17/2011/. 10.1007/s10072-011-0622-4.

[3] A. Fago, F. B. Jensen, B. Tota et al., “Integrating nitric oxide, nitrite and hydrogen sulfide signaling in the physiological adaptations to hypoxia: a comparative approach,” Comparative Biochemistry and Physiology—A Molecular and Integrative Physiology, vol. 162, no. 1, pp. 1–6, 2012. 10.1016/j.cbpa.2012.01.011.

[4] Wei Guo, Ze-yu Cheng and Yi-zhun Zhu, “Hydrogen sulfide and translational medicine,” Acta Pharmacologica Sinica volume 34, pages 1284–1291 (2013). 10.1038/aps.2013.127.

[5] Yi-Hong Liu, Chang-Dong Yan, and Jin-Song Bian, “Hydrogen sulfide: a novel signaling molecule in the vascular system,” Journal of Cardiovascular Pharmacology, 01/2011. 10.1097/FJC.0b013e31820eb7a1.

[6] Yuan Zhang, Zhi-Han Tang, Zhong Ren, Shun-Lin Qu, Mi-Hua Liu, Lu-Shan Liu and Zhi-Sheng Jiang, “Hydrogen sulfide, the next potent preventive and therapeutic agent in aging and age-associated diseases,” Molecular and Cellular Biology Feb 2013, 33 (6) 1104–1113. 10.1128/MCB.01215-12.

[7] Yong-Peng Yu, Zhen-Guang Li, Dao-Zhen Wang, Xia Zhan, Jing-Hua Shao. “Hydrogen sulfide as an effective and specific novel therapy for acute carbon monoxide poisoning,” Biochemical and Biophysical Research Communications, 2011. 10.1016/j.bbrc.2010.11.113.

## 1. Introduction

Nitric oxide (NO) and carbon monoxide (CO) are established physiologic messenger molecules, and the former serving as an endothelial cell-derived relaxing factor (EDRF) has an important role in the regulation of blood pressure [1]. Even though hydrogen sulfide (H_2_S) has long been known as a noxious and toxic gas, recent accumulated evidence suggests that H_2_S, as an important endogenous vasodilator and neuromodulator [2, 3], has been implicated in similar functions [4]. A physiologic role for H_2_S in regulating blood pressure, its potent neuroprotective [5], and anti-inflammatory effects support a hypothesis that H_2_S might act as an effective agent that may have therapeutic potential against brain damage induced by oxidative stress, inflammation, hypoxic vasoconstriction, and other factors. Brain damage mainly induced by cerebral vasospasm is a potentially incapacitating or lethal complication in patients with aneurysmal SAH. Thus, the development of effective preventative and therapeutic interventions is an urgent and significant need. The objective of this paper is to present an overview of the pathogenesis of brain injury after SAH and the multiple physiologic functions of H_2_S in the vascular system, based on which a hypothesis is providing that H_2_S might be an effective therapy agent for brain injury after SAH.

## 2. Distribution and Level of H_2_S in Vascular Tissues

There are three known enzymes that produce H_2_S endogenously in mammalian tissue: cystathionine-synthase (CBS), gamma lyase (CGL or cystathionine gamma-lyase, CSE), and 3-mercaptopyruvate sulfur transferase (3MST). There are three major fates of H_2_S in the body. First, most of the H_2_S produced in the body is oxidized in the mitochondria to an end product of sulfate. The remaining H_2_S either is methylated by thiol S-methyltransferase (TSMT) to methanethiol and dimethyl sulfide or binds to methemoglobin to form sulfhemoglobin. In most tissues, CBS and CSE, which are responsible for catalyzing the production of H_2_S, are both pyridoxal-5-phosphate-dependent enzymes that utilize cysteine and homocysteine as substrates to liberate ammonium, pyruvate, and H_2_S [6]. It was originally believed that CBS was responsible for H_2_S production in the brain through the activation of the Ca2+/calmodulin pathway. H_2_S is produced by 3MST from l-cysteine and alpha-ketoglutarate through the metabolism with cysteine aminotransferase (CAT) [7].

H_2_S production was observed in ileum, portal vein, and thoracic aorta homogenates when L-Cys and PLP were administered. Moreover, the application of aminooxyacetate (CBS inhibitor) inhibited H_2_S production in ileum but failed to affect generation of H_2_S in portal vein and thoracic aorta, suggesting the lack of CBS in vascular tissues [8, 9]. Many studies have been focused on this issue [3, 10–13]. Endogenous H_2_S has been paid more and more attention to since its physiological discovery. It is expected that physiological concentration of H_2_S may vary extensively from synthesizing enzymes in different tissues. It was reported that endogenous concentration of H_2_S in rat, human, and bovine brains ranged within 50–160 *μ*M [14–16], whereas its serum concentration was about to be 50 *μ*M. Previous study showed that H_2_S does not circulate in the plasma at high enough concentration to be detectable in blood and plasma from a variety of animals, including trout, mouse, Wistar rat, Dawley rat, pig, and cow [11]. H_2_S concentration in brain and liver homogenates was measured 14 ± 3 nM and 17 ± 3 nM, respectively [12].

## 3. Multiple Physiologic Functions and Beneficial Effects of H_2_S in Biological Systems

The biological effects of H_2_S on vascular system have been studied for more than a decade. It was first reported that H_2_S concentration dependently relaxed norepinephrine precontracted portal vein and thoracic aorta, and this relaxation effect was reversible upon removal of chemicals [8]. This vasodilatory effect was later found to be present not only in thoracic aorta, but also in other types of vascular tissues including mesenteric arteries, pulmonary artery, and tail artery. H_2_S induced vasorelaxation is mainly brought about by the opening of KATP channels [17, 18]. Other signaling mechanisms for the vasorelaxant effect of H_2_S may involve depletion of intracellular ATP levels in aortic rings [19] and intracellular acidosis [20]. KATP channels are likely not to be involved in H_2_S induced vasoconstriction [21]. However, many studies were performed to attempt to unveil the underlying mechanism of H_2_S induced constrictive effects. Firstly, H_2_S may react with NO to form a compound, which by itself has no effect on vascular contractility. NO, as a potent physiological vasodilator, quenched by H_2_S, which might underlie H_2_S induced vascular constriction response [19, 22]. Moreover, H_2_S exerted inhibitory effects on endothelial nitric oxide synthase (eNOS) activity [18]. H_2_S was found to downregulate cAMP levels in vascular smooth muscle cells (SMC) [21]. H_2_S exerts antihypertensive effects to different extents in different hypertensive models. The antihypertensive role of H_2_S is confirmed. Slow H_2_S-releasing compounds and therefore might be as potential therapeutics for hypertension treatment in the future. The data mentioned above suggests that the evidence for H_2_S's effects on vascular smooth muscle tone is contradictory.

The mechanisms by which H_2_S affects injured cells are complicated. Interestingly, in some studies, H_2_S induced proinflammatory effects which indicates that the background of these immunomodulatory influences still remains elusive [19]. H_2_S has several effects on mitochondria of cardiac cells such as the reversible inhibition of cytochrome C oxidase, which leads to preservation of mitochondrial structure and function after ischemia/reperfusion. Inhibition of mitochondrial respiration in the injured myocytes results in attenuated generation of reactive oxygen species (ROS) and may alter the function of the affected cell [22]. H_2_S decreases lipid peroxidation by scavenging hydrogen peroxide and superoxide in a model of isoproterenol induced myocardial injury. Besides these mechanisms, H_2_S also acts as a direct scavenger neutralizing cytotoxic reactive species like peroxynitrite.

The physiological actions of H_2_S make this gas ideally suited to protect the heart, brain, liver, kidney, and lungs against injury during ischemia/reperfusion (I/R). In recent years, the cytoprotective effects of endogenous and exogenous H_2_S have been investigated in models of in vitro and in vivo ischemic injury [6]. Previous study showed that either endogenous or exogenous increases in H_2_S at the time of reperfusion limit the extent of myocardial infarction, which was accompanied by a decrease in myocardial inflammation and mitochondrial function preservation [23]. Regulation and cytoprotective, physiological, and chemical roles of H_2_S in biological systems including ischemic injury of cardiac-cerebral vascular disease have been investigated in many studies [23–30].

The vasodilatory effect of H_2_S, which might be a potential candidate to be considered as EDRF and was later found to be present in widespread arteries including thoracic aorta, mesenteric arteries, pulmonary, and other types of vascular tissues [30], was particularly involved at the microcirculation level [31, 32]. Based on the accumulated data, several signaling pathways including cyclic adenosine monophosphate (cAMP)/protein kinase A (PKA), NO/cyclic guanosine monophosphate (cGMP)/protein kinase G (PKG) [33, 34], and calcium/calmodulin (CaM) [35] were involved in the vasoregulatory effect of H_2_S. Previous study also suggested that hypoxia and H_2_S shared a common and unique pathway in the excitation-contraction process. The inhibition of H_2_S synthesis can inhibit both hypoxic vasoconstriction and hypoxic vasodilation [36].

This role of H_2_S on smooth muscle is likely to be a general effect. Besides the recently established vasoregulatory role of H_2_S, its anti-inflammatory effect in different systems has been investigated. Recent studies have demonstrated that H_2_S may in fact limit inflammation and free radical damage [37], while another study indicated that H_2_S might exert an important proinflammatory role in regulating the severity of pancreatitis and associated lung injury [36]. Previous data suggested that ROS were implicated not only in the control of vascular tone in blood, but also in the H_2_S-induced regulatory function of vascular tissues [33, 38]. These findings raise the possibility that pharmacologic enhancement of H_2_S formation could be an alternative approach for repairing neuron damage induced by vasospasm, ischemia, oxidative stress, inflammation, and other factors, while previous study has demonstrated that H_2_S treatment improved endothelium-dependent coronary microvascular relaxation, providing biochemical myocardial protection via attenuation of caspase-independent apoptosis and autophagy in the experimental model [39]. It suggested that the evidence for H_2_S's effects on autophagy might be contradictory.

## 4. Causes and Mechanisms of Brain Injury after Subarachnoid Hemorrhage

Accumulated data indicate that not only delayed ischemic injury [40], which had been considered the most important cause of poor outcome after SAH, but also early brain injury has become the vital determinant of the intensity of later developing neurological complications [41]. Delayed cerebral vasospasm that develops 3–7 days after SAH had traditionally been considered the most important determinant of delayed ischemic injury. In recent years, increasing evidence suggests that several mechanisms, including ionic and physiological, biochemical, molecular, persistent vascular changes, cell death, oxidative stress, and inflammatory cascade activation, were involved in the pathogenesis of early brain injury after SAH [42–44]. Previous papers show that many experimental studies as well as autopsies performed on the brains of patients after SAH demonstrated extensive ischemic damage. Many factors including elevation of intracranial pressure (ICP), release of vasoactive substances during erythrocyte lysis, platelet aggregation, lipid peroxidation, unopposed sympathetic activity, and alterations in the nitric oxide/nitric oxide synthase (NO/NOS) pathways may contribute to this brain injury [45].

Though cerebral vasospasm probably plays some part in brain injury after SAH, neurologic injury may not be entirely explained by ischemia. The relationship between cerebral vasospasm and neurologic outcome might be associated with other coexisting causative factors such as microvascular dysfunction and complex neuronal-glial interactions. Previous study suggested that cerebral infarction contributed to poor outcome by vasospasm-independent effects after SAH [44, 46]. Cortical spreading depression (CSD) as one of the interesting mechanisms in brain damage after SAH has gained increasing attention [47, 48]. It has been previously reported that leukocyte-endothelial cell interactions, which played a significant role in the pathophysiology of cerebral vasospasm, could explain the clinical variability and time course of this disease [49]. Therefore, timely therapeutic targeting of the inflammatory response may prevent vasospasm-related brain damage and improve outcomes in patients with SAH.

Autophagy, being a self-degradative process, which plays a housekeeping role in removing misfolded or aggregated proteins, clearing damaged organelles, and eliminating intracellular pathogens [50, 51], is important for balancing sources of energy at critical times in development and in response to nutrient stress. The autophagy pathway has been reported to be involved in several central nervous system diseases such as cerebral ischemia [52], hypoxia-ischemia induced brain injury, and traumatic brain injury [53]. In the experimental SAH model, it is suggested that autophagy activation could participate in the pathogenesis of early brain injury induced by SAH. That is to say, activation of the autophagy pathway may play a potential role to attenuate the development of brain damage in SAH [51].

## 5. Involvement of H_2_S in Vascular Relaxation

H_2_S, known as a poisonous and toxic gas of the rotten egg, is endogenously produced from the metabolism of L-cysteine through constitutively expressed enzymes (including CBS and CSE) [54, 55]. H_2_S produced by CSE can enhance the outward flux of K+ by opening the KATP channel, resulting in membrane potential hyperpolarization and vascular smooth muscle relaxation [17, 56]. The vascular effects of H_2_S are thought to be partially mediated by functional endothelium [57]. It is also suggested to coordinate with NO to regulate smooth muscle tone [8]. Previous studies have reported that CSE knockout mice displayed significant hypertension and reduce endothelium-dependent vasodilation, while reducing H_2_S levels in many tissues including serum, heart, and aorta [58]. As an H_2_S donor, intravenous delivery of NaHS can temporarily reduce systolic blood pressure in mice, suggesting that H_2_S plays a crucial role in regulating physiological vasodilation and blood pressure.

The effect of H_2_S on the regulation of vascular smooth muscle tone and blood pressure suggests that it may be involved in the regulation of cerebral blood flow cerebral vasospasm after SAH and may have potential therapeutic effects in preventing and reversing cerebral vasospasm. Although there is limited evidence for H_2_S and cerebral vasospasm after SAH, more research is needed in future basic research.

## 6. The Effect of NO in Pathophysiology, Reversal of Delayed Cerebral Vasospasm

As the most famous EDRF, NO is produced by eNOS in the intima and neuronal nitric oxide synthase (nNOS) and inducible nitric oxide synthase (iNOS) in the outer membrane of cerebrovascular and smooth muscle cells [59, 60]. Other substances that exhibit the same characteristics of EDRF include CO [61] and H_2_S [62].

H_2_S, NO, and nitrite are formed in vivo and are of vital importance in the tissue response to hypoxia. These signaling molecules are involved in a multitude of processes including the regulation of vascular tone, cellular metabolic function, and cytoprotection [63].

NO can directly activate soluble guanosine cyclase (sGC) to catalyze the conversion of GTP to circular GMP in smooth muscle cells. An increase in circulating GMP levels leads to the prevention of Ca2+-dependent activation of myosin light chain kinase [64]. It has been suggested that activation of Ca2+-dependent KATP channels [65, 66] is associated with NO-induced relaxation of vascular smooth muscle. In addition to causing vasodilation, reducing vascular resistance, NO derived from endothelial cells can reduce smooth muscle proliferation and inhibit platelet aggregation and adhesion [67].

In animals suffering from SAH, oxyhemoglobin (oxyHb), which is gradually released from the blood clot of the subarachnoid space by erythrocyte lysis, is an effective NO scavenger [68]. Together with vasoconstrictors such as endothelin-1 (ET-1), cyclooxygenase (COX) products, and ROS [69], the bioavailability of nitric oxide derived from the endothelium, neurons, and nitrosated nerves will be reduced. Excessive production of NO acts under pathological conditions such as inflammation and cerebral ischemia, resulting in the production of peroxynitrite and other highly toxic compounds. This may partly explain the ischemic neurological deficit after subarachnoid hemorrhage. Similarly, previous studies have shown that inhibition of iNOS may result in a treatment that reduces ischemic brain damage [70]. On the other hand, eNOS-mediated pretreatment may have a beneficial effect on reducing vasospasm and cerebral ischemia after SAH [71]. Therefore, based on these data, the primary therapeutic goal of cerebral vasospasm after SAH is to exogenously administrating NO donors, inhibiting PDE and BOXs, and prevention of oxyHb neurotoxicity [72]. In animal models, intravenous administration of nitroglycerin (NTG) or sodium nitroprusside (SNAP) in the form of nitrate is effective in preventing cerebral vasospasm [73,74]. Nitrite is an endogenous NO donor in the blood [75], which may provide a means of overcoming the reduction of NO production after SAH [72]. In the primate SAH model, intravenous administration of sodium nitrite for 2 weeks prevented the development of vasospasm without altering blood pressure, suggesting that nitrite can locally release NO in the subarachnoid space [76]. However, further studies of nitrite are needed to elucidate the pharmacokinetics of sodium nitrite in humans and to establish appropriate doses and safety. Local (intracranial/intracranial arterial) delivery of NO donors is clinically unattractive due to increasing the risk of serious complications or surgical approaches [72].

## 7. The Parallels and Contrastive Effects between NO and H_2_S: Looking for Crosstalk

NO and H_2_S are ancient, prebiotic, and chemically reactive molecules, which living organisms have evolved to cope with and ultimately make use of for signaling purposes. The reactivity of NO and H_2_S is controlled partly by keeping their steady-state levels in vivo rather low (in the nanomolar to low micromolar range) by balancing consumption with production, by the NOS enzymes in the case of NO, and by the enzymes CSE, CBS, or the tandem enzymes cysteine aminotransferase (CAT) and 3-mercaptopyruvate sulfurtransferase (3-MST) in the case of H_2_S. Another common feature of these enzymatic pathways is that both have an amino acid as substrate: L-Arg is the substrate for NOS and L-Cys is a substrate for CSE and CBS [63]. In the vertebrate circulation, NO and H_2_S diffuse away from their site of production and react specifically with their biological targets. NO may activate soluble guanylate cyclase present in adjacent smooth muscle cells and trigger vasorelaxation or react with ROS to form secondary reactive nitrogen oxide species (e.g., peroxynitrite). Conversely, H_2_S may activate potassium channels and, depending on whether the vessel is systemic or pulmonary/gill, may then trigger vasorelaxation or vasoconstriction, respectively, or even both at different time scales [77]. Thus, whereas NO is strictly a vasodilator, H_2_S may mediate dilation and/or constriction. The prevailing view is that both NO and H_2_S have a limited radius of effects and have mainly paracrine/autocrine actions. NO, however, can be transported in the blood as plasma and erythrocyte nitrite and S-nitrosothiols, which are relatively stable oxidation products of NO metabolism. Such products may then be recycled back to NO at distant locations and therefore NO can be also regarded as an endocrine signaling molecule. Such endocrine function has not been demonstrated for H_2_S up to now [63]. Furthermore, it is not yet clear to which extent H_2_S is capable of generating S-nitrosothiols or other related products. It appears conceivable that a possible crosstalk between the two pathways might involve some thiol-based biochemical compounds common to either pathway [78]. On the other hand, H_2_S relaxations are reported to be independent of NO synthesis or cGMP [79], which suggests other downstream targets specific for H_2_S. The antithrombotic effect of hydrogen sulfide is partly mediated by an upregulation of nitric oxide synthases [80]. Previous study reported that H_2_S affects [Ca2+]i homeostasis which is mediated by H_2_S-evoked NO production [81]. These hypotheses, while pointing to a possible delicate balance between these two pathways, need to be confirmed experimentally.

Clearly, understanding the crosstalk between these two pathways is a major challenge for future investigations. Whereas highly sensitive and specific reductive chemiluminescence has been essential to determine type, concentrations, and fate of NO metabolites, concerning H_2_S detection in vivo, intracellular H_2_S concentrations are not known due to the lack of a suitable, sensitive, and specific method to analyze its various forms currently. As it is the case for NO that can be regenerated from nitrite, there is no evidence that H_2_S metabolites can be transported in the blood, stored in tissues, and reactivated to generate H_2_S at present. It is possible therefore that our understanding on this aspect may change with the development of alternative methodologies in the future. There is a need for future research in this field to better understand the complexity of biological interaction between the H_2_S and NO/nitrite signaling pathways.

## 8. H_2_S: A Novel Neuroprotectant for Central Nervous System Diseases Based on Its Role in Physiology

Since 1996, when H_2_S was firstly reported, the role of H_2_S has been gradually revealed by various contributions worldwide [82]. H_2_S has been generally recognized as an important signaling molecule in cardiovascular and nervous systems. The physiological effects of H_2_S have been confirmed by understanding the therapeutic implications of H_2_S in central nervous system (CNS) related diseases including neurodegenerative diseases, diabetes, and cancer, among others. In the CNS, H_2_S ameliorates ischemic injuries but leads to the aggravation of stroke. H_2_S concentration enhancement could induce cerebral infarct, while CBS or CSE inhibitors can reverse this effect on the brain. The role of H_2_S in neuron protection has been shown in glutamate-induced death with enhancement of cysteine and *γ*-glutamylcysteine concentrations, which leads to increasing concentrations of GSH [83–86].

Parkinson's disease (PD) is a common neurodegenerative disease with various manifestations, among which is cognitive deficiency, namely, dementia, which is characterized by progressive loss of dopaminergic neurons in the substantia nigra (SN). In a PD rat model induced by 6-hydroxydopamine (6-OHDA), the endogenous H_2_S level significantly decreased in the SN. However, H_2_S treatment can specifically inhibit accumulation of proinflammatory factors in the striatum and 6-OHDA-evoked NADPH oxidase activation, oxygen consumption, and microglial activation in the SN [87]. Dementia is usually ascribed to changes in the nucleus basalis of Meynert and the cerebral cortex [88]. H_2_S has also been suggested to attenuate vascular dementia injury via inhibition of apoptosis by regulating Bcl-2 and Bax expressions [89].

Alzheimer's disease (AD) is the most common form of dementia, which is pathologically characterized by the accumulation of senile plaques containing activated microglia and amyloid beta peptides (A-beta) [90]. As mentioned above, endogenous H_2_S is predominantly produced in the brain from cysteine by CBS. In the brains of AD patients, lower levels of H_2_S are a strong risk factor for the development of AD [91, 92]. Localized increases in H_2_S could delay aggravation and exacerbation of symptoms in patients with AD [85, 93, 94]. In addition, patients with Down syndrome overproduce H_2_S due to high level of the urinary excretion of thiosulfate, suggesting a positive relationship between H_2_S concentration and the aggravation of this disease [94–96]. Accordingly, it has been demonstrated that H_2_S has protective effects against A-beta-induced cell injury by inhibiting inflammation, promoting cell growth, and preserving mitochondrial function [97]. Moreover, H_2_S can protect neurons from oxidative stress, which is responsible for neuronal damage and degeneration in AD. H_2_S protects neurons against glutamate-mediated oxidative stress by enhancing the activities of *γ*-GCS and cystine transport, which results in increasing glutathione levels [98]. Neurotoxicity of elevated Hcy is associated with inhibition of endogenous H_2_S generation. It has been suggested that H_2_S could reduce neurotoxicity induced by Hcy and that enhancement of H_2_S synthesis may be a useful therapeutic strategy against Hcy-induced AD [99]. These lines of evidences suggest that H_2_S is a promising therapeutic target for treating neurodegenerative diseases.

## 9. Hypothesis and Theoretical Aspects Fundamental to This Hypothesis

At present, the mainstay treatment of brain injury after SAH is neurocritical care management aimed at reducing secondary brain injury, oral nimodipine, hemodynamic therapy, statin/magnesium/nicardipine therapy, cerebrospinal fluid (CSF) drainage, and endovascular techniques to improve cerebral vasospasm. Most of these treatments are aimed at one or two physiological and pathological mechanisms of brain injury after SAH. Then, a therapy or therapies focused on multiple mechanisms may prevent the brain injury and improve the long-term outcome of SAH better. In fact, the possible roles and target of endogenous H_2_S in pathophysiological regulation of SAH have not been investigated. In light of the multiple physiologic roles and beneficial effects of H_2_S in mammalian tissue mentioned above, our hypothesis is that H_2_S might act as an effective agent which might provide a novel approach to the treatment of brain injury after SAH. This hypothesis is based on the following facts: it is suggested that vascular contractility is regulated by endogenous and exogenous H_2_S at physiologically relevant concentrations [17]. Furthermore, some studies reported that vasorelaxation response elicited by H_2_S was greater in small mesenteric arteries as compared to that in larger vascular tissues such as aorta [100]. H_2_S is an endogenous substance that is also produced, reaching an endogenous level of 50–160 *μ*M. Cerebral vascular smooth muscle cells would be exposed to significant amounts of H_2_S [58, 101]. Previous study indicated that exogenous H_2_S, generated as sodium sulfide, could limit the inflammatory response to acute myocardial I/R injury in an animal model [102].

It has been known that the mechanism underlying the brain injury after SAH is interlaced with multiple causative and/or pathogenic factors, including free radicals reactions, inflammatory processes, apoptosis, an imbalance between vasoconstrictor and vasodilator substances (endothelium derived substances, NO, endothelin, arachidonic acid metabolites, etc.), an upheaval of factors which regulate vascular tone, and endothelial proliferation [103]. As mentioned above, autophagy could participate in the pathogenesis of brain injury induced by SAH, which will provide novel ideas for pursuing therapeutic agents for SAH-induced brain injury. The success of these current therapies in reducing incidence of cerebral vasospasm without reduction in brain injury and improved quality of life indicates that treating vasospasm alone may not achieve favorable result. Therefore, therapies against SAH designed to direct towards inhibiting brain injury may prove more beneficial in preventing neurological deterioration.

## 10. Conclusion

In light of a wide range of physiological roles of H_2_S as mentioned above, it produces physiological and pathological functions in many organs and systems. Previous papers have reported that H_2_S could protect against reperfusion injury, lethal hypoxia, and exerted anti-inflammatory and antiapoptotic activities and oxidative stress effects [104] as well as effect of autophagy activation. Furthermore, as an endogenous gasotransmitter and a potential treatment, H_2_S has distinct advantages over pharmaceutical drugs: its tissue compatibility and high blood-brain barrier permeability are stronger than many other antioxidants and it is an endogenous substance. We speculate that H_2_S could be a potentially effective approach to the treatment of brain injury including vasospasm after SAH. With our existing knowledge about the beneficial effects of H_2_S, the future of H_2_S as a potential therapy against brain injury after SAH is deemed to be promising and exciting.

## 11. Looking Forward: Challenges for Translation of the Toxic Molecule H_2_S and Its Therapeutic Application

Investigation of the role of H_2_S is still in its infancy. H_2_S has both scientific and technological values. The latter tends to dominate because of its financial value and direct applicability. The scientific investigation of H_2_S is also important for the elucidation of its base fundamental roles. Certainly, problems and challenges will arise and the limitations of experimental materials will constrain further research in the field of H_2_S. A sustained and controlled H_2_S-releasing donor that functions both in vitro and in vivo has not been found yet. With an increased understanding of the various H_2_S mechanisms in the body, further study of H_2_S becomes more difficult and complicated. H_2_S is known as a third gaseous signaling molecule, which means that it plays the role of a messenger. The concentration of H_2_S has been proven to have relevance in particular diseases; for instance, it is overproduced in sepsis and found at inadequate levels in AD [105]. Therefore, the mechanism controlling the actual concentration of H_2_S in certain tissues may become the ultimate problem for H_2_S-related research. It should be emphasized that a relevant relationship does not mean a relationship of causation. By regulating the H_2_S concentration in particular tissues, symptoms of a specific disease can be controlled, which implies that the origin of the disease has not been addressed. That is to say, the regulation of H_2_S can only provide transient protection from certain diseases, such as hypertension. The challenges of the sustained and controlled release of H_2_S-releasing drugs were mentioned above. Another difficulty in H_2_S related research comes from the multiple functions of H_2_S, which cause a shortage of specific effects which are dose-, time-, and tissue-dependent.

The pathophysiology of cerebral vasospasm after SAH is complex and needs further clarification. The role of NO in regulating cerebral blood flow, pathogenesis, and treatment of cerebral vasospasm as well as delayed ischemic neurological deficits after SAH has been extensively studied. As one of other EDRFs, H_2_S may provide potential targets for the development of preventive and therapeutic measures, including cerebral vasospasm. The interaction between these factors also needs to be studied. The inflammatory response associated with SAH may represent a key pathway in the pathogenesis of cerebral vasospasm and delayed ischemic neurological deficits [106, 107]. The role of EDRF, such as H_2_S, is needed to be elucidated in regulating cerebral blood flow and cerebral vasospasm, preventing neuroischemic defects. For patients with different diseases, H_2_S may need to be administered as different drugs. Therefore, a focus on the general effects of H_2_S, such as on brain injury after SAH, is rational.

  References
(1) Ndisang JF, Tabien HEN, Wang R. Carbon monoxide and hypertension. Journal of Hypertension. 2004; 22(6):1057–1074.
(2) Li Y-F, Xiao C-S, Hui R-T. Calcium sulfide (CaS), a donor of hydrogen sulfide (H2S): a new antihypertensive drug? Medical Hypotheses. 2009; 73(3):445–447.
(3) Szabõ C. Hydrogen sulphide and its therapeutic potential. Nature Reviews Drug Discovery. 2007; 6(11):917–935.
(4) Fiorucci S, Antonelli E, Distrutti E, et al. Inhibition of hydrogen sulfide generation contributes to gastric injury caused by anti-inflammatory nonsteroidal drugs. Gastroenterology. 2005; 129(4):1210–1224.
(5) Kimura Y, Dargusch R, Schubert D, Kimura H. Hydrogen sulfide protects HT22 neuronal cells from oxidative stress. Antioxidants and Redox Signaling. 2006; 8(3-4):661–670.
(6) Nicholson CK, Calvert JW. Hydrogen sulfide and ischemia-reperfusion injury. Pharmacological Research. 2010; 62(4):289–297.
(7) Shibuya N, Tanaka M, Yoshida M, et al. 3-Mercaptopyruvate sulfurtransferase produces hydrogen sulfide and bound sulfane sulfur in the brain. Antioxidants and Redox Signaling. 2009; 11(4):703–714.
(8) Hosoki R, Matsuki N, Kimura H. The possible role of hydrogen sulfide as an endogenous smooth muscle relaxant in synergy with nitric oxide. Biochemical and Biophysical Research Communications. 1997; 237(3):527–531.
(9) Chen P, Poddar R, Tipa EV, et al. Homocysteine metabolism in cardiovascular cells and tissues: implications for hyperhomocysteinemia and cardiovascular disease. Advances in Enzyme Regulation. 1999; 39:93–109.
(10) Li L, Moore PK. Putative biological roles of hydrogen sulfide in health and disease: a breath of not so fresh air? Trends in Pharmacological Sciences. 2008; 29(2):84–90.
(11) Whitfield NL, Kreimier EL, Verdial FC, Skovgaard N, Olson KR. Reappraisal of H2S/sulfide concentration in vertebrate blood and its potential significance in ischemic preconditioning and vascular signaling. The American Journal of Physiology—Regulatory Integrative and Comparative Physiology. 2008; 294(6):R1930–R1937.
(12) Furne J, Saeed A, Levitt MD. Whole tissue hydrogen sulfide concentrations are orders of magnitude lower than presently accepted values. The American Journal of Physiology—Regulatory Integrative and Comparative Physiology. 2008; 295(5):R1479–R1485.
(13) Levitt MD, Abdel-Rehim MS, Furne J. Free and acid-labile hydrogen sulfide concentrations in mouse tissues: anomalously high free hydrogen sulfide in aortic tissue. Antioxidants and Redox Signaling. 2011; 15(2):373–378.
(14) Abe K, Kimura H. The possible role of hydrogen sulfide as an endogenous neuromodulator. Journal of Neuroscience. 1996; 16(3):1066–1071.
(15) Savage JC, Gould DH. Determination of sulfide in brain tissue and rumen fluid by ion-interaction reversed-phase high-performance liquid chromatography. Journal of Chromatography—Biomedical Applications. 1990; 526(2):540–545.
(16) Wang R. The gasotransmitter role of hydrogen sulfide. Antioxidants and Redox Signaling. 2003; 5(4):493–501.
(17) Cheng Y, Ndisang JF, Tang G, Cao K, Wang R. Hydrogen sulfide-induced relaxation of resistance mesenteric artery beds of rats. The American Journal of Physiology—Heart and Circulatory Physiology. 2004; 287(5):H2316–H2323.
(18) Kubo S, Doe I, Kurokawa Y, Nishikawa H, Kawabata A. Direct inhibition of endothelial nitric oxide synthase by hydrogen sulfide: contribution to dual modulation of vascular tension. Toxicology. 2007; 232(1-2):138–146.
(19) Kiss L, Deitch EA, Szabó C. Hydrogen sulfide decreases adenosine triphosphate levels in aortic rings and leads to vasorelaxation via metabolic inhibition. Life Sciences. 2008; 83(17-18):589–594.
(20) Lee SW, Cheng Y, Moore PK, Bian J-S. Hydrogen sulphide regulates intracellular pH in vascular smooth muscle cells. Biochemical and Biophysical Research Communications. 2007; 358:1142–1147.
(21) Jia JL, Liu Y-H, Khin ESW, Bian J-S. Vasoconstrictive effect of hydrogen sulfide involves downregulation of cAMP in vascular smooth muscle cells. The American Journal of Physiology—Cell Physiology. 2008; 295(5):C1261–C1270.
(22) Li L, Rose P, Moore PK. Hydrogen sulfide and cell signaling. Annual Review of Pharmacology and Toxicology. 2011; 51:169–187.
(23) Popov D. An outlook on vascular hydrogen sulphide effects, signalling, and therapeutic potential. Archives of Physiology and Biochemistry. 2013; 119:189–194.
(24) Stein A, Bailey SM. Redox biology of hydrogen sulfide: implications for physiology, pathophysiology, and pharmacology. Redox Biology. 2013; 1:32–39. [PMC free article]
(25) Snijder PM, de Boer RA, Bos EM, et al. Gaseous hydrogen sulfide protects against myocardial ischemia-reperfusion injury in mice partially independent from hypometabolism. PLoS ONE. 2013; 8e63291
(26) Kimura H. Production and physiological effects of hydrogen sulfide. Antioxidants & Redox Signaling. 2013; 20(5):783–793.
(27) Du JT, Li W, Yang JY, Tang CS, Li Q, Jin HF. Hydrogen sulfide is endogenously generated in rat skeletal muscle and exerts a protective effect against oxidative stress. Chinese Medical Journal. 2013; 126:930–936.
(28) Bos EM, Wang R, Snijder PM, et al. Cystathionine *γ*-lyase protects against renal ischemia/reperfusion by modulating oxidative stress. Journal of the American Society of Nephrology. 2013; 24:759–770.
(29) Yin J, Tu C, Zhao J, et al. Exogenous hydrogen sulfide protects against global cerebral ischemia/reperfusion injury via its anti-oxidative, anti-inflammatory and anti-apoptotic effects in rats. Brain Research. 2013; 1491:188–196.
(30) Zhou J, Wu PF, Wang F, Chen JG. Targeting gaseous molecules to protect against cerebral ischaemic injury: mechanisms and prospects. Clinical and Experimental Pharmacology and Physiology. 2012; 39:566–576.
(31) Han J, Chen ZW, He GW. Acetylcholine and sodium hydrosulfide-induced endothelium-dependent relaxation and hyperpolarization in cerebral vessels of global cerebral ischemia-reperfusion rat. Journal of Pharmacological Sciences. 2013; 121:318–326.
(32) Di Villa Bianca RD, Sorrentino R, Coletta C, et al. Hydrogen sulfide-induced dual vascular effect involves arachidonic acid cascade in rat mesenteric arterial bed. Journal of Pharmacology and Experimental Therapeutics. 2011; 337(1):59–64.
(33) Muzaffar S, Shukla N, Bond M, et al. Exogenous hydrogen sulfide inhibits superoxide formation, NOX-1 expression and Rac1 activity in human vascular smooth muscle cells. Journal of Vascular Research. 2008; 45(6):521–528.
(34) Bucci M, Papapetropoulos A, Vellecco V, et al. Hydrogen sulfide is an endogenous inhibitor of phosphodiesterase activity. Arteriosclerosis, Thrombosis, and Vascular Biology. 2010; 30(10):1998–2004.
(35) Bauer CC, Boyle JP, Porter KE, Peers C. Modulation of Ca2+ signalling in human vascular endothelial cells by hydrogen sulfide. Atherosclerosis. 2010; 209(2):374–380.
(36) Olson KR, Dombkowski RA, Russell MJ, et al. Hydrogen sulfide as an oxygen sensor/transducer in vertebrate hypoxic vasoconstriction and hypoxic vasodilation. Journal of Experimental Biology. 2006; 209(20):4011–4023.
(37) Bhatia M, Wong FL, Fu D, Lau HY, Moochhala SM, Moore PK. Role of hydrogen sulfide in acute pancreatitis and associated lung injury. The FASEB Journal. 2005; 19(6):623–625.
(38) Liu Y-H, Bian J-S. Bicarbonate-dependent effect of hydrogen sulfide on vascular contractility in rat aortic rings. The American Journal of Physiology—Cell Physiology. 2010; 299(4):C866–C872.
(39) Osipov RM, Robich MP, Feng J, et al. Effect of hydrogen sulfide on myocardial protection in the setting of cardioplegia and cardiopulmonary bypass. Interactive Cardiovascular and Thoracic Surgery. 2010; 10(4):506–512.
(40) Pluta RM, Hansen-Schwartz J, Dreier J, et al. Cerebral vasospasm following subarachnoid hemorrhage: time for a new world of thought. Neurological Research. 2009; 31(2):151–158.
(41) Alaraj A, Charbel FT, Amin-Hanjani S. Peri-operative measures for treatment and prevention of cerebral vasospasm following subarachnoid hemorrhage. Neurological Research. 2009; 31(6):651–659.
(42) Friedrich V, Flores R, Muller A, Sehba FA. Escape of intraluminal platelets into brain parenchyma after subarachnoid hemorrhage. Neuroscience. 2010; 165(3):968–975.
(43) Sehba FA, Pluta RM, Zhang JH. Metamorphosis of subarachnoid hemorrhage research: from delayed vasospasm to early brain injury. Molecular Neurobiology. 2011; 43(1):27–40.
(44) Spallone A, Acqui M, Pastore FS, Guidetti B. Relationship between leukocytosis and ischemic complications following aneurysmal subarachnoid hemorrhage. Surgical Neurology. 1987; 27(3):253–258.
(45) Sehba FA, Bederson JB. Nitric oxide in early brain injury after subarachnoid hemorrhage. Acta neurochirurgica. Supplement. 2011; 110(1):99–103.
(46) Vergouwen MDI, Ilodigwe D, MacDonald RL. Cerebral infarction after subarachnoid hemorrhage contributes to poor outcome by vasospasm-dependent and -independent effects. Stroke. 2011; 42(4):924–929.
(47) Bosche B, Graf R, Ernestus R-I, et al. Recurrent spreading depolarizations after subarachnoid hemorrhage decreases oxygen availability in human cerebral cortex. Annals of Neurology. 2010; 67(5):607–617.
(48) Dreier JP, Major S, Manning A, et al. Cortical spreading ischaemia is a novel process involved in ischaemic damage in patients with aneurysmal subarachnoid haemorrhage. Brain. 2009; 132(7):1866–1881.
(49) Pradilla G, Chaichana KL, Hoang S, Huang J, Tamargo RJ. Inflammation and cerebral vasospasm after subarachnoid hemorrhage. Neurosurgery Clinics of North America. 2010; 21(2):365–379.
(50) Glick D, Barth S, Macleod KF. Autophagy: cellular and molecular mechanisms. Journal of Pathology. 2010; 221(1):3–12.
(51) Wang Z, Shi X-Y, Yin J, Zuo G, Zhang J, Chen G. Role of autophagy in early brain injury after experimental subarachnoid hemorrhage. Journal of Molecular Neuroscience. 2012; 46(1):192–202.
(52) Liu C, Gao Y, Barrett J, Hu B. Autophagy and protein aggregation after brain ischemia. Journal of Neurochemistry. 2010; 115(1):68–78.
(53) Zhang YB, Li SX, Chen XP, et al. Autophagy is activated andmight protect neurons fromdegeneration after traumatic brain injury. Neuroscience Bulletin. 2008; 24:143–149.
(54) Wagner F, Asfar P, Calzia E, Radermacher P, Szabó C. Bench-to-bedside review: hydrogen sulfide–the third gaseous transmitter: applications for critical care. Critical Care. 2009; 13(3):p. 213.
(55) Mancardi D, Penna C, Merlino A, Del Soldato P, Wink DA, Pagliaro P. Physiological and pharmacological features of the novel gasotransmitter: hydrogen sulfide. Biochimica et Biophysica Acta. 2009; 1787(7):864–872.
(56) Dominy JE, Stipanuk MH. New roles for cysteine and transsulfuration enzymes: production of H2S, a neuromodulator and smooth muscle relaxant. Nutrition Reviews. 2004; 62(9):348–353.
(57) Zhao W, Wang R. H2S-induced vasorelaxation and underlying cellular and molecular mechanisms. The American Journal of Physiology—Heart and Circulatory Physiology. 2002; 283(2):H474–H480.
(58) Yang G, Wu L, Jiang B, et al. H2S as a physiologic vasorelaxant: hypertension in mice with deletion of cystathionine *γ*-lyase. Science. 2008; 322(5901):587–590.
(59) Daneshtalab N, Smeda JS. Alterations in the modulation of cerebrovascular tone and blood flow by nitric oxide synthases in SHRsp with stroke. Cardiovascular Research. 2010; 86(1):160–168.
(60) Bredt DS. Endogenous nitric oxide synthesis: biological functions and pathophysiology. Free Radical Research. 1999; 31(6):577–596.
(61) Kanu A, Whitfield J, Leffler CW. Carbon monoxide contributes to hypotension-induced cerebrovascular vasodilation in piglets. The American Journal of Physiology—Heart and Circulatory Physiology. 2006; 291(5):H2409–H2414.
(62) Zoccali C, Catalano C, Rastelli S. Blood pressure control: hydrogen sulfide, a new gasotransmitter, takes stage. Nephrology Dialysis Transplantation. 2009; 24(5):1394–1396.
(63) Fago A, Jensen FB, Tota B, et al. Integrating nitric oxide, nitrite and hydrogen sulfide signaling in the physiological adaptations to hypoxia: a comparative approach. Comparative Biochemistry and Physiology—A Molecular and Integrative Physiology. 2012; 162(1):1–6.
(64) Moncada S, Palmer RMJ, Higgs EA. Nitric oxide: physiology, pathophysiology, and pharmacology. Pharmacological Reviews. 1991; 43(2):109–142.
(65) Plane F, Wiley KE, Jeremy JY, Cohen RA, Garland CJ. Evidence that different mechanisms underlie smooth muscle relaxation to nitric oxide and nitric oxide donors in the rabbit isolated carotid artery. British Journal of Pharmacology. 1998; 123(7):1351–1358.
(66) Kinoshita H, Ishikawa T, Hatano Y. Role of K+ channels in augmented relaxations to sodium nitroprusside induced by mexiletine in rat aortas. Anesthesiology. 2000; 92(3):813–820.
(67) Toda N, Ayajiki K, Okamura T. Cerebral blood flow regulation by nitric oxide: recent advances. Pharmacological Reviews. 2009; 61(1):62–97.
(68) Martin W, Villani GM, Jothianandan D, Furchgott RF. Selective blockade of endothelium-dependent and glyceryl trinitrate-induced relaxation by hemoglobin and by methylene blue in the rabbit aorta. Journal of Pharmacology and Experimental Therapeutics. 1985; 232(3):708–716.
(69) Toda N, Okamura T. The pharmacology of nitric oxide in the peripheral nervous system of blood vessels. Pharmacological Reviews. 2003; 55(2):271–324.
(70) Iadecola C, Zhang F, Casey R, Nagayama M, Elizabeth Ross M. Delayed reduction of ischemic brain injury and neurological deficits in mice lacking the inducible nitric oxide synthase gene. Journal of Neuroscience. 1997; 17(23):9157–9164.
(71) Vellimana AK, Milner E, Azad TD, et al. Endothelial nitric oxide synthase mediates endogenous protection against subarachnoid hemorrhage-induced cerebral vasospasm. Stroke. 2011; 42(3):776–782.
(72) Pluta RM. Dysfunction of nitric oxide synthases as a cause and therapeutic target in delayed cerebral vasospasm after SAH. Neurological Research. 2006; 28(7):730–737.
(73) Sun B-L, Zhang S-M, Xia Z-L, et al. L-arginine improves cerebral blood perfusion and vasomotion of microvessels following subarachnoid hemorrhage in rats. Clinical Hemorheology and Microcirculation. 2003; 29(3-4):391–400.
(74) Ito Y, Isotani E, Mizuno Y, Azuma H, Hirakawa K. Effective improvement of the cerebral vasospasm after subarachnoid hemorrhage with low-dose nitroglycerin. Journal of Cardiovascular Pharmacology. 2000; 35(1):45–50.
(75) Cosby K, Partovi KS, Crawford JH, et al. Nitrite reduction to nitric oxide by deoxyhemoglobin vasodilates the human circulation. Nature Medicine. 2003; 9(12):1498–1505.
(76) Pluta RM, Dejam A, Grimes G, Gladwin MT, Oldfield EH. Nitrite infusions to prevent delayed cerebral vasospasm in a primate model of subarachnoid hemorrhage. Journal of the American Medical Association. 2005; 293(12):1477–1484.
(77) Dombkowski RA, Russell MJ, Schulman AA, Doellman MM, Olson KR. Vertebrate phylogeny of hydrogen sulfide vasoactivity. The American Journal of Physiology—Regulatory Integrative and Comparative Physiology. 2005; 288(1):R243–R252.
(78) Whiteman M, Li L, Kostetski I, et al. Evidence for the formation of a novel nitrosothiol from the gaseous mediators nitric oxide and hydrogen sulphide. Biochemical and Biophysical Research Communications. 2006; 343(1):303–310.
(79) Olson KR, Whitfield NL. Hydrogen sulfide and oxygen sensing in the cardiovascular system. Antioxidants and Redox Signaling. 2010; 12(10):1219–1234.
(80) Kram L, Grambow E, Mueller-Graf F, Sorg H, Vollmar B. The anti-thrombotic effect of hydrogen sulfide is partly mediated by an upregulation of nitric oxide synthases. Thrombosis Research. 2013; 132:e112–e117.
(81) Moustafa A, Habara Y. Hydrogen sulfide regulates Ca2+ homeostasis mediated by concomitantly produced nitric oxide via a novel synergistic pathway in exocrine pancreas. Antioxidants & Redox Signaling. 2013; 20(5):747–758.
(82) Chen Y-H, Yao W-Z, Geng B, et al. Endogenous hydrogen sulfide in patients with COPD. Chest. 2005; 128(5):3205–3211.
(83) Ishigami M, Hiraki K, Umemura K, Ogasawara Y, Ishii K, Kimura H. A source of hydrogen sulfide and a mechanism of its release in the brain. Antioxidants and Redox Signaling. 2009; 11(2):205–214.
(84) Cunha TM, Dal-Secco D, Verri WA, Jr., et al. Dual role of hydrogen sulfide in mechanical inflammatory hypernociception. European Journal of Pharmacology. 2008; 590(1–):127–135.
(85) Liu Y-Y, Sparatore A, Del Soldato P, Bian J-S. ACS84, a novel hydrogen sulfide-releasing compound, protects against amyloid *β*-induced cell cytotoxicity. Neurochemistry International. 2011; 58(5):591–598.
(86) Qu K, Lee SW, Bian JS, Low C-M, Wong PT-H. Hydrogen sulfide: neurochemistry and neurobiology. Neurochemistry International. 2008; 52(1):155–165.
(87) Hu L-F, Lu M, Tiong CX, Dawe GS, Hu G, Bian J-S. Neuroprotective effects of hydrogen sulfide on Parkinson's disease rat models. Aging Cell. 2010; 9(2):135–146.
(88) Korczyn AD. Vascular contribution to dementia in Parkinson's disease. Neurodegenerative Diseases. 2010; 7(1–3):127–130.
(89) Zhang L-M, Jiang C-X, Liu D-W. Hydrogen sulfide attenuates neuronal injury induced by vascular dementia via inhibiting apoptosis in rats. Neurochemical Research. 2009; 34(11):1984–1992.
(90) Hampel H. Amyloid-beta and cognition in aging and Alzheimer's disease: molecular and neurophysiological mechanisms. Journal of Alzheimer's Disease. 2013; 33:S79–S86.
(91) Seshadri S, Beiser A, Selhub J, et al. Plasma homocysteine as a risk factor for dementia and Alzheimer's disease. New England Journal of Medicine. 2002; 346(7):476–483.
(92) Tang X-Q, Shen X-T, Huang Y-E, et al. Hydrogen sulfide antagonizes homocysteine-induced neurotoxicity in PC12 cells. Neuroscience Research. 2010; 68(3):241–249.
(93) Whiteman M, Winyard PG. Hydrogen sulfide and inflammation: the good, the bad, the ugly and the promising. Expert Review of Clinical Pharmacology. 2011; 4(1):13–32.
(94) Tan BH, Wong PT-H, Bian J-S. Hydrogen sulfide: a novel signaling molecule in the central nervous system. Neurochemistry International. 2010; 56(1):3–10.
(95) Ufnal M, Sikora M, Dudek M. Exogenous hydrogen sulfide produces hemodynamic effects by triggering central neuroregulatory mechanisms. Acta Neurobiologiae Experimentalis. 2008; 68(3):382–388.
(96) Wang M-J, Cai W-J, Li N, Ding Y-J, Chen Y, Zhu Y-C. The hydrogen sulfide donor NaHS promotes angiogenesis in a rat model of hind limb ischemia. Antioxidants and Redox Signaling. 2010; 12(9):1065–1077.
(97) Liu Y-Y, Bian J-S. Hydrogen sulfide protects amyloid-*β* induced cell toxicity in microglia. Journal of Alzheimer's Disease. 2010; 22(4):1189–1200.
(98) Kimura Y, Kimura H. Hydrogen sulfide protects neurons from oxidative stress. The FASEB Journal. 2004; 18(10):1165–1167.
(99) Tang X-Q, Shen X-T, Huang Y-E, et al. Inhibition of endogenous hydrogen sulfide generation is associated with homocysteine-induced neurotoxicity: role of ERK1/2 activation. Journal of Molecular Neuroscience. 2011; 45(1):60–67.
(100) Wang R. Hydrogen sulfide: a new EDRF. Kidney International. 2009; 76(7):700–704.
(101) Rochette L, Vergely C. Hydrogen sulfide (H2S), an endogenous gas with odor of rotten eggs might be a cardiovascular function regulator. Annales de Cardiologie et d'Angeiologie. 2008; 57(3):136–138.
(102) Sodha NR, Clements RT, Feng J, et al. Hydrogen sulfide therapy attenuates the inflammatory response in a porcine model of myocardial ischemia/reperfusion injury. Journal of Thoracic and Cardiovascular Surgery. 2009; 138(4):977–984.
(103) Kolias AG, Sen J, Belli A. Pathogenesis of cerebral vasospasm following aneurysmal subarachnoid hemorrhage: putative mechanisms and novel approaches. Journal of Neuroscience Research. 2009; 87(1):1–11.
(104) Yu Y-P, Li Z-G, Wang D-Z, Zhan X, Shao J-H. Hydrogen sulfide as an effective and specific novel therapy for acute carbon monoxide poisoning. Biochemical and Biophysical Research Communications. 2011; 404(1):6–9.
(105) Gong Q-H, Shi X-R, Hong Z-Y, Pan L-L, Liu X-H, Zhu Y-Z. A new hope for neurodegeneration: possible role of hydrogen sulfide. Journal of Alzheimer's Disease. 2011; 24(2):173–182.
(106) Ostrowski RP, Colohan AR, Zhang JH. Molecular mechanisms of early brain injury after subarachnoid hemorrhage. Neurological Research. 2006; 28(4):399–414.
(107) Dumont AS, Dumont RJ, Chow MM, et al. Cerebral vasospasm after subarachnoid hemorrhage: putative role of inflammation. Neurosurgery. 2003; 53(1):123–135.
